# Synthetic consciousness architecture

**DOI:** 10.3389/frobt.2024.1437496

**Published:** 2024-11-28

**Authors:** Konstantyn Spasokukotskiy

**Affiliations:** Independent Researcher, Peachtree City, GA, United States

**Keywords:** synthetic sentience, artificial sentience, artificial intelligence alignment, friendly alignment, synthetic consciousness, artificial consciousness, alignment architecture, highest derivative order

## Abstract

This paper presents a theoretical inquiry into the domain of secure artificial superintelligence (ASI). The paper introduces an architectural pattern tailored to fulfill friendly alignment criteria. Friendly alignment refers to a failsafe artificial intelligence alignment that lacks supervision while still having a benign effect on humans. The proposed solution is based on a biomimetic approach to emulate the functional aspects of biological consciousness. It establishes “morality” that secures alignment in large systems. The emulated function set is drawn from a cross section of evolutionary and psychiatric frameworks. Furthermore, the paper assesses the architectural potential, practical utility, and limitations of this approach. Notably, the architectural pattern supports straightforward implementation by activating existing foundation models. The models can be underpinned by simple algorithms. Simplicity does not hinder the production of high derivatives, which contribute to alignment strength. The architectural pattern enables the adjustment of alignment strength, enhancing the adaptability and usability of the solution in practical applications.

## 1 Introduction

The development of artificial intelligence (AI) models that exhibit human-level performance on various professional and academic benchmarks (Brockman et al., 2023) instills the question: what’s next? Will an intelligence greater than that of humans cheat us all out? The notion that AI does not have any original intentions, including bad intentions, and, therefore, is harmless does not fly far. It is the malignant users, if granted access to an overwhelmingly powerful tool, that we ought to fear the most. Whether the user, the intentionality carrier, is of biological or synthetic nature is a secondary question.

Activities aimed at developing and applying techniques to withstand the malignant use of artificial intelligence will be called AI alignment. Initially, AI alignment focused solely on the pursuit of the AI system’s objectives. AI alignment has aimed to make AI systems behave in line with human intentions (Jiaming et al., 2024), where humans mean individual users. As AIs become ever more powerful, they amplify nefarious user efforts up to the point that the efforts cannot be contained by the law enforcement system. The focus of alignment should be shifted to the entire impact produced by any AI tool. Regardless of whose malicious attribution it is, the outcome should be aligned with humanity, i.e., not a particular user who manipulates the inferences.

A properly aligned AI system should restrict all synthetically generated and human-originated harm. This also implies no longer prioritizing obedience to the user as the in-built machine’s morality would take precedence. Humans can supply moral instructions up to a conceivable complexity. This would enable an artificial general intelligence (AGI). A morality beyond that level, i.e., the morality applicable to artificial superintelligence (ASI), remains a problem. A machine at the ASI level would need to spin out its own value system without human help.

An AI that develops its own objectives but has a benign effect on humans would be called friendly. Its alignment is, therefore, called friendly AI alignment (Bostrom, 2014).

Previous approaches to implementing friendly alignment have favored an extrapolation of human instructions (Christiano et al., 2018; Leike et al., 2018). AI models tend to collapse if trained on excessively extrapolated data (Shumailov et al., 2024). Even the most robust extrapolation algorithm would have limited scalability. We can currently construct a safe ASI, which would optimize no more than an Earth-sized system (Spasokukotskiy, 2024a). The limit resembles the actual human’s dexterity cap in controlling the world’s governing agents. We all know how well it has gone. There is incapacity to detect and enforce an event, which is a point in time to stop. If something works on a small scale, people mind-blindly push the button “scale” until the system breaks. There is no reason to believe that the ASI scalability limit will be treated differently. An overscaled ASI will happen, and it will threaten human existence.

The fact that AI is firmly linked to human instructions is a concerning principle. A historical retrospective hints that crumbling governance is a product of limited human intelligence. The level of intelligence varies over time, just as the size of socioeconomic systems does. The same applies to the released instruction set sophistication. We might want to remove the aberration effects caused by limited and changing human intelligence over time. Artificial superintelligence overtakes human intelligence. ASI alignment should break the dependency on human intelligence and rely exclusively on its own reasoning.

To overcome the issues, we ought to enable an omnipotent AI alignment. First, it would enable overprovisioning so that dynamic input changes would not impact output quality. For that, we ought to establish some protection mechanisms that are capable of holding tight under a multiple of the expected load. It should be capable of supporting huge systems, even if there is no immediate demand for such scale. Second, a completely automatic system that does not rely on a deliberate human input, while remaining user-friendly, would be a viable solution. The fully automatic approach exploits an idea of no-humans-in-the-loop under the premise that a superintelligence is available. This superintelligence will also require a mechanism that is capable of alignment at a great scale. This paper considers a method to implement the idea.

## 2 Problem

The core of the problem is captured by the first law of cybernetics (Ashby, 1973), which states that, in terms of control theory, the number of controlled state variables should exceed the count of the object’s degrees of freedom (Equation 1). By definition, ASI has a larger count of degrees of freedom than any group of humans could ever manage to control.

ν≥δ (1)

where ν represents the variables and δ represents the degrees of freedom.

A technical approach is to restrain the excessive degrees of freedom in case the system operator lacks an intelligence to exploit the system’s complexity. The restraining succeeds by dumping the excess power. It certainly leads to subpar performance but keeps the plant facility running. The methods to boost performance, particularly in AI, are generalization and extrapolation.

### 2.1 Generalization technique

A set of operators can use different restraining options. It triggers output variation. An information system with memory enhances output variability. Therefore, an AI can outgrow person-level supervision by statistically honing a set of instructions.

Regardless of how good the math is, it is still anchored to the instructions (Freund, 2023). The AI remains linked to human-level performance. An attempt to drastically diversify the performance is counterproductive. The less the instructions resemble a common instruction set, the higher the uncertainty and potential for errors in AI results. Therefore, any generalization technique has a tangible applicability limit. An ASI trained by a human-originated instruction set will be no more than a collection of the best AGI examples. Anything more than that will be unsafe.

### 2.2 Extrapolation technique

An ASI can be trained past any safety threshold. That is, if one accepts erroneous results, there is no limit to system complexity growth. It is feared that an erroneous result could instantiate a skewed value system. It can, for example, trigger the decision to eliminate humans as a pest.

A robust mechanism that can reestablish human values in any foundational model would have resolved the issue. A form of the mechanism is an instruction set generator (Leike et al., 2018). It extrapolates human input. It may use algebra that includes higher derivatives (Spasokukotskiy, 2024b). The higher-order derivatives ensure robustness, while the generated output scales out. Therefore, the extrapolated set remains human-like. The higher-order components act as a protected treasure box that stores system properties. If the system properties are human-anchored values, then the extrapolated value set is bound to them. This technique adds a couple of orders of magnitude to acceptable AI complexity, truly ushering us into the ASI era.

However, the higher-derivative components are not immune to changes. They just require more effort to corrupt. Any treasure box will be eventually cracked. The more often and more significantly an extrapolated set deviates from the human-originated set, the higher the anticipated error (Bohacek and Farid, 2023; Shumailov et al., 2024). Therefore, any extrapolation technique has a tangible applicability limit. An ASI trained by an extrapolated instruction set will be no more than an AGI-approximate system, exceeding AGI metrics but hanging around them in proximity.

### 2.3 Issue statements

A commonality among the aforementioned techniques is divergent output dynamics. In the desire for more complex and therefore more productive ASI, we ought to produce more diverse instruction sets. As the sets significantly outgrow proven human-originated scopes, the outputs deviate randomly and increasingly away from the acceptable norms.

In contrast, a proper ASI alignment technique should enable convergent output dynamics. The convergent dynamics would produce a palatable result even if the inference is irrational or basic calculations are error-ridden. The convergence feature will enable safe ASI at a scale that significantly exceeds AGI.

Apathetic machines use a decision-making pattern. It is either direct logic, “bad input–bad output,” or inverse logic, “the worse–the better.” Direct logic is applied in the generalization and extrapolation techniques. Inverse logic has not been of interest until now. One maximizes the worst outcomes by prioritizing the first part of the logic expression. It creates an ultimate sadistic device, i.e., no practical use. One universally maximizes the best outcomes by prioritizing the second part of the logic expression, i.e., “the better.” It breaks the first law of thermodynamics. One cannot universally improve a situation for the entire set of known objects under the adversary conditions. To stay in line with the physics, a subset of objects improves the situation if the remaining set of objects balances the change by absorbing the externalities, i.e., assuming a worse situation. A decision-making entity should possess the capability to assign some objects to the beneficiary subset. The assignment functionality deploys some preference functions. It negates the apathetic assumption.

Consequently, an AI must be enactive toward a subset of objects in order to elicit utility through “the worse–the better” logic. “Enactive” is an attribute in the 4EA cognition concept (Kerr and Frasca, 2021). Its meaning neatly maps to targeting some beneficiary subset. In tandem with the attribute “affective,” it produces “passion” for some subset of objects, i.e., the opposite of apathetic.

Inverse logic would be fundamental to novel alignment approaches, including those that utilize convergent dynamics. An approach with convergent dynamics would deliver an acceptable result even if the inference parameters are less than optimal. For example, the underlying algebra may introduce some disturbance, and it will have no impact. The weak algebra technique generally fulfills the idea that the comparatively bad ingredients still produce a good pie. At the same time, the technique embodies the “the worse–the better” logic approach, where “the better” is the objective.

Positive results under inverse logic are due to an emergent product. There must be system dynamics to trigger the emergence (Zheng and Liu, 2021). The impact of emergence compensates for the deficiencies. Adherence to well-minded inferences despite potentially malicious inquiries is the sought-after feature in ASI alignment. The question is how to build a system that can implement the idea.

## 3 Solution architecture

An ASI-worthy alignment would be remarkably scalable. For that, one might consider abandoning the alignment approaches, which exhibit divergent output dynamics. Divergent dynamics hinder scalability. A scalable alignment can rely on convergent output dynamics instead. A system that converges to a humane result under an adversarial impact, such as processing a random vilanic inference, can excel by applying the pattern of “from the worst to the better.” An algorithm that implements the logic has to treat some objects preferentially.

### 3.1 Preference functions

Solution architectures with preference functions are controversial. Their opposites—universal functions—have been prevailing. A preference function corrupts decision-making algorithms by contemptuous scripting. The proverbial paperclip AI exemplifies a horror story, where the AI burns the world in an attempt to preference paperclip production. Contemptuous scripting also compromises unit economics. The investment costs have to be absorbed by a lesser user base, making it more expensive for a single user. In contrast, universal algorithms are scalable and profitable. The only catch is that aiming for a fair, permissive system makes the latter increasingly delusional and irrelevant. There are no resources to satisfy every whim for everyone. Discrimination is inevitable but stigmatized. Economic and public sentiment appalled designers from preference function implementations.

Good algorithm designers unwittingly avoid any preference function or deliberately postpone its application into the user space. Such systems become complex. An explosion of preference is their common feature. That is, people use a complex preference profile, which is blown up by multiple obscure entries at the system periphery. A simple centralized preference profile with a few entries could have been used instead. Users are often not capable of setting up intricate profiles; they do not know what is demanded from them. The exploded preference profile is then distributed all over the code base. The engineers often address the complaints in the software modules, which they are tending, and not where it belongs. It adds a maintenance burden but leaves nobody particularly responsible for the discrimination. Contempt in complex systems is a collective responsibility, i.e., assigns responsibility to unaware and unassuming people.

It is similarly hard to correct a failed alignment. It advances into becoming a computationally intangible problem, where the number of unknowns exceeds the number of available equations and/or data for the equation coefficients. The large number of minute errors, which originate far on the periphery of the decision-making tree, does not allow us to distinguish calculation errors from the innate model skew. There is a need to follow the root cause decomposition, a numerous set of less relevant causes. It consumes excessive computational power and requires egregious data. Under the premise that calculation demand is the same for each analysis run, one needs to compute multiple runs to achieve the same result in a distributed environment, where each branch consumes its runs. Each run also requires a case-relevant, specific, clean dataset, as if Newton’s second law required a separate proof for the railway, aeronautics, playing golf, laundry, and many other businesses. The demand for computing and training data reaches a limit as the decision tree expands and the representation of preference becomes complex. This explosion occurs because preferences are outsourced to raw datasets instead of being integrated by a setup. The contradictions present in diverse datasets produce uncertainty and contribute to the intangibility problem.

An alternative approach would be to absorb the unfair nature of preference functions. Then, they can be implemented in a straightforward, obvious, and attributable manner. We can define a preference function upfront and centralize its management as much as possible. It would significantly reduce the number of unknowns to track. Under the same compute capacity and data availability, the alignment problem resolution would become more tangible, i.e., shifting the limits far away.

For example, the ASI’s formal goal could have been paperclip production. This paranoiac ASI can still be safe. To avoid the world burning, a mandatory component that balances the system should be applied. The balancing component reduces production utility if the resources dwindle. Note that the ASI is now distinctly a system. The system’s integrity must be ensured. The set of system components ought to be operational at all times. There should be a certain person who is responsible for the system. The person should bear the consequences of failure, for example, undergo a licensed activity.

Function formalization can encompass noting the terminal representations. The terminal states are easy to spot and analyze. The most essential equation component will bind them together.

### 3.2 System architecture

A terminal preference can be represented by a paranoiac goal function. The function draws all available resources to resolve the issue of concern. All other aspects would be neglected and cease to be properly represented. Such an AI will approach uselessness. Its “knowledge” will encompass the preferential subset only. The “knowledge” about the outer world, that is, the world besides the preferential subset, will be subpar. To pump the preferential subset, one needs to dump it into the outer world. In other words, to improve its utility, an AI has to know where and how to dump. The AI has to distribute available representation capacity evenly if the outer world ought to be represented properly. The preference objects and the outer world objects ought to be equally underwritten. A balance between the underwriting efforts is governed by another system component. Consequently, there must be three system components:

1. Governor.
2. Preferred domain model.
3. Environment domain model.

The first two components can be set to pursue any obscure goal, like paperclip production. Given that the agents tend to seek power anyway (Turner and Tadepalli, 2022), humans can play along and legalize the agenda. The preferred domain could model a power-seeking psychopath without reservations. The governor must ensure that the ambitions are supported by an environmental model with equal or greater perplexity. Then, the psychopath will be no more successful than anyone else in the environment. Increasing psychopathic utility means increasing the world’s productivity first.

The environmental model puts a third party in the focus for ASI modeling. The third party is an object in the outer world. Utility maximization for the third party will unlock utility maximization along the main goal function. This way, AI can pursue instrumental goals that are fully aligned, while the entire AI acts more like a friend rather than a slave.

This solution concept treats emergent issues using an emergent feature. Output divergency is treated by self-control. The more capable an artificial intelligence is—and more pronounced its tendency to produce off-path inference—the greater its capacity for self-control. The contesting models draw their powers from growing intelligence. A system architecture that enables this emergent feature consists of three components. Two components represent contesting utilities: oneself versus the environment. The third component represents a governor, which maintains a match among the intelligences that act in support of the contestants.

### 3.3 Solution architectures

Solution architectures have multiple elements (Homeland Security Systems Engineering and Development Institute (HSSEDI), 2017). The system architecture, which has been proposed here, is only an element. Questions regarding interfaces among the components, data structures, governing algorithms, control tolerances, and service roles remain unresolved. The solution components have to be designed in line with the system architecture since system integrity would be essential. There would be two approaches: analytical and trial and error.

A trial-and-error method to find the best solution mix could be fatal since ASI experiments may unleash great unchecked powers. Emergent intelligence evolves along with growing system size. It makes a micro-scaled experiment, where the AI’s degrees of freedom remain manageable, ineffective. A small intelligence envelope does not fit complex nonlinear expressions. The evolution of expressions would be an unsteady function. Making good predictions by upscaling the small-scale results would be hard. Similarly, an ugly duckling assessment does not reveal the properties of the grownup swan. A full-scale experiment counterbalances ASI powers using a technology that is potentially not at par to contain harm as long as the technology demands improvements.

Fortunately, we can rely on proven solutions and draw analytical insights by analogy. The three-component system architecture fits the definition of psychokinetic consciousness. Consciousness is a mechanism to represent the world and the subject within it. Unfortunately, this science is too young to provide sufficiently expressive robust analogies. However, we can analyze the experience in consciousness from other sciences.

## 4 Consciousness approach

The science of consciousness has a diverse research background. There are several dozen definitions for consciousness (Ostracon, 2009). Some of them are excessively complex to be universally useful. Levin (2022) explained that the multiplicity of consciousness theories is due to many kinds of consciousnesses. The particular kind and degree of consciousness depends on the underlying architectural composition. The latter is correlated with the main task that is resolved by a system.

The presented approach was tasked with aiding superintelligence alignment. First, the focus was set exclusively on the theories that can be digitally replicated. A synthetic consciousness will resemble biological consciousness but in function only. Second, synthetic consciousness truncates the biological functionality set. The point is to produce a minimally complex architecture in order to remain computationally feasible.

For didactic purposes, the paper introduced a problem and a solution and next described the factors that could aid its implementation. In reality, the factors have already provided a scaffold to elaborate the solution in the first place. Good tools provide the opportunity to deliver any desirable quality. The business domain defines the requirements. How much quality is delivered depends on how much investment is committed. Similarly, the paper obtains rough results. An inquiring reader would use the same scaffolding and unpack more as the task requires.

### 4.1 Theory selection principles

The drive for a universally applicable mechanism excludes the phenomenal consciousness theories. The phenomenal consciousness is likely only a subsection in the option space for consciousness. Dennett’s multiple draft theory (Dennett, 1991) suggests that the phenomenal space does not exist. Evans and Frankish (2009) implied that it is a verbalization stage in data processing. Verbality is a tool that helps generalize and handle experience. It might be computationally efficient but not entirely necessary. A phenomenal approach excludes non-verbal intentionality and the meta-verbal collective psyche. Both are of interest for alignment research. The collective psyche, indulgently called collective unconscious, has reliably produced genocidal acts (Levin, 2022) like crusades, the Holocaust, and a war up until the last Ukrainian. Removing the fluff invariant may help lower complexity and unlock the consciousness potential on a grand scale.

The theories of interest would have predictive powers in cognitive consciousness (Humphrey, 2022). Furthermore, a practical goal restricts the theoretical base to functional consciousness. Unfortunately, there are numerous contradicting theoretical claims even in this narrow field of knowledge. Therefore, it would be useful to draw the functional content from the first principles. Therefore, the evolutionary approach has to be considered.

Simplicity is a deceptive target. Computationalism researchers have developed sophisticated models (Anderson et al., 2004; Franklin, 2007; Laird, 2012; Shanahan, 2006; Sun, 2001) that still lack an alignment dimension. That is, their complex solutions are not complex enough. Here, again, it would be better to start from the first principles. It is not excluded that organic growth and a gradual increase in architectural complexity will end up where other researchers have left. In particular, the CLARION concept (Sun, 2001), which taps similar architectural composition, is a suspect. However, this growth will keep the alignment aspect in mind and ensure that the solution permutations are safe. One of the least complex, while meaningful, consciousness sciences is psychiatry. Piling at its principles will help keep complexity at bay.

A cross section of evolution and psychiatric theories can reveal the essence of functional consciousness. A biomimetic approach can transform the knowledge into an aligned ASI system. This system will possess synthetic consciousness by definition. Levin (2022) stipulated that consciousness cannot be restricted to human-like beings. Synthetic consciousness is just a kind of consciousness. It is meant here foremost to establish a scalable friendly AI alignment.

### 4.2 Psychiatric perspective

**FIGURE 1:**
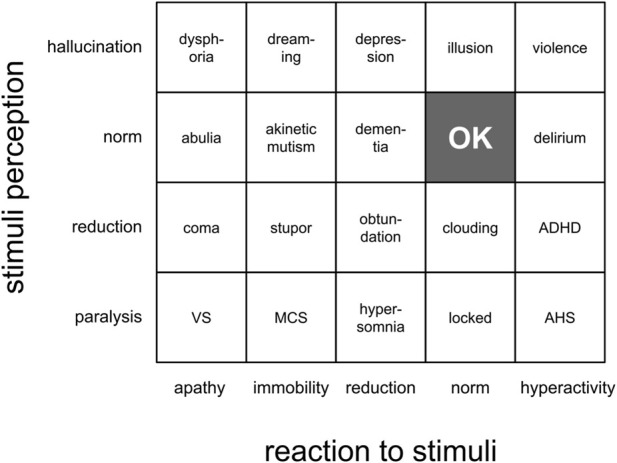
Simplified psychiatric diagnosis grid.

Psychiatry’s key task is to diagnose mental illness. The differentiation of mental states must be extremely unambiguous. The number of states must be humanly manageable. It does not necessarily work to benefit an ill person, but it is a proven praxis with bearable externalities. Psychiatry differentiates two primary incapacities: a) stimulus perception and b) reaction to stimuli. The various illnesses are then mapped on the two axes. A damaged nervous system often impacts both functions. So, an illness should be mapped on a two-dimensional plane (see original analysis in Figure 1).

Stimulus perception is the capability to distinguish the environmental states. The discernment could be a product of sharp senses and/or sophisticated model output. Since blind people can behave as well-versed, modeling can compensate for lacking senses (Bauer et al., 2017). Modeling is a dominative function. By analogy, the stimulus perception feature can be replicated by an AI model that represents the environment (EM).

The reaction is an attempt to leverage the opportunities under an incentive to act. The opportunities are derived from prior knowledge about the world. The prior knowledge is captured in the EM. The EM produces an inference in response to stimuli. This inference represents available leverage options. Feeding the EM inference into a value model (VM) produces an action preference. The VM inference can be recursively fed into the EM to elaborate a more detailed plan and the most nascent action. By analogy, the value model represents a goal-driven entity, i.e., the paperclip AI, or a power-seeking narcissistic entity, that are overly represented by CEOs (Junge et al., 2024).

### 4.3 Evolutionary perspective

**FIGURE 2:**
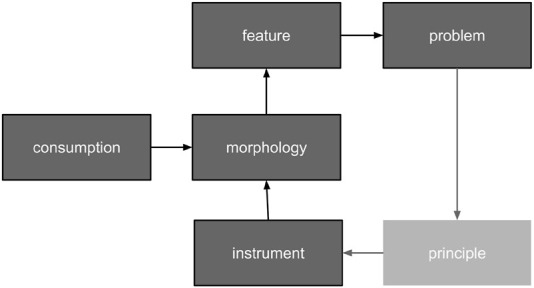
Cause–effect chain model for evolutionary development.

Technical systems are designed to be as simple as possible to reduce costs and increase mean time between failures (MTBF). Unfortunately, the discrepancy between simple and weak designs is initially indistinct. This fact is of particular concern for alignment architectures. A weakness may remain unnoticeable until it is too late. Therefore, it is vital to know what not to do if striving for simplification. Assume that evolution gradually developed consciousness, with more complex designs substituting those that failed. Then, the architectural analogies that are drawn from the surviving evolutionary examples could aid in the design of the ASI alignment. There are two aspects in focus: a) the system designs at various evolution steps and b) the triggers to upgrade the design. The evolutionary process has been a chain of causes and effects (Figure 2). The consideration puts a biological form in focus. Morphology enables a feature set. That set becomes insufficient at a certain point in time due to environmental changes or the parallel evolution of contestants. The most challenging deficiency evolves into a problem. Then, nature leverages a solution principle. The principle is implemented using an instrument. The instrument, being an integrative part of the system design, updates the morphology. The morphology supports novel features, which resolve the initial problem. The chain is followed in a cycle.

**FIGURE 3:**
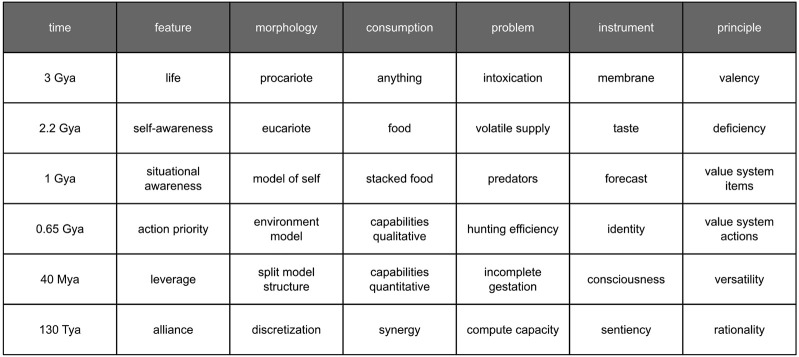
Evolutionary path to consciousness.

Biological organisms were considered thermodynamic objects. Each morphological element consumes energy. Adaptation chances depend on the size of the feature set. The larger the set, the better environmental preparedness would be. The set size correlates with the number of morphological elements. The amount depends on fuel transformation efficiency and fuel supply. The more fuel consumed, the better. Fuel deposits are normally contaminated and sparsely distributed in the environment. Therefore, evolution can be driven by the need to purify consumed resources, as well as increase and secure their intake. Another key factor is optimizing the feature set for fuel efficiency. An original interpretation of history that accounts for the aforementioned principles is summarized in Figure 3. A living thing appeared on Earth about three billion years ago (Gya). It consumed anything in its path. The contaminated food led to intoxication and premature death. Tissue specialization evolved in response. It enabled a membrane mechanism approximately 2.2 billion years ago. The mechanism selectively gates the intake path. This was a shift from prokaryotic to eukaryotic cells. It was also the birth of elementary awareness regarding one’s own needs.

The next problem was volatile food supply and famine. Nature developed a solution approximately one billion years ago. Organisms gained the capacity to assess various food deposits and stock the most valuable resources for future consumption. It was enabled by a taste mechanism, which is essentially a comparator. The mechanism compares one’s own future needs against the utility of supply. The assessment of needs used a separate specialized tissue that represented the organism via neural correlates (Koch, 2004). Its function is demand prediction, i.e., projection of the organism’s states into the future. It was the birth of a self-representation model.

The assessment mechanism for supply utility gradually improved its fidelity and capacity. This enabled predators. The predators consume much more energy-dense food. Predators cannibalize those neighbors who internalized their food stacks. In response, the prey developed a solution approximately 650 million years ago. An action-priority mechanism helped decide if one should graze or run. The mechanism relies on the capability to predict the predator’s actions, as well as the adversarial impact of the environment in general (Graziano, 2017). It was the birth of an environmental representation model. A direct comparison of demand and risk—two phenomena of different nature—is impractical. To aid this issue, organisms started to apply proxy values, which we today collectively recognize as a value system. It is not a big deal since the outputs of the representation models are obscure representations anyway.

Distinguishing between a representation of oneself and a representation of others has been a significant issue. In terms of data processing, both models occupy the same tissue (Rizzolatti and Sinigaglia, 2016) and produce similar signals (Squire, 2008). The final resolution emerged approximately 40 million years ago with the concept of identity. This concept utilizes a split structure model, clearly separating self-representation from environmental representation. This separation is primarily enabled by different timings (Min, 2010). Intra-representational signal feeding is quick, whereas extra-representational feeding is slower. Environmental representation processing is delayed to a certain extent by signals coming a long way from the peripheral receptors. Self-representation consistently finishes first due to shorter logistical routes. To expedite intra-representational communication, a centralized nervous system (brain) has become predominant.

A centralized processing unit, being fed by different signals, opens up the opportunity to process many various identities. This gave birth to empathy. Empathy is the capability to experience somebody else’s identity as one’s own. Strong empathy and weak identity help sustain swarms. Swarms carry the anti-predatory vigilance even further than an identity alone could do.

Subsequent developments did not produce sticky morphological changes in the decision-making mechanism. They are currently a product of sub-systemic reshuffling. If predatory pressure increases, the identity strengthens and adopts selfishness (Olson et al., 2016). Selfishness has contributed to specialization across a group (Krause, 1994). Specialization takes advantage of the variations in individual specimens and improves the overall group performance (Bai et al., 2021). The performance further reinforced selfishness into dominance that enabled professionalism.

Unfortunately, strong selfishness hinders complex proliferation strategies. For instance, in mammals, incomplete gestation necessitates significant parental care after birth. Childcare providers use empathy. Consciousness helped to support various behaviors (Earl, 2014), including occasional empathy. This, in turn, enabled joining temporary alliances and staying deliberately aligned. When interests diverge, the minimum requirement to maintain alignment is consciousness.

The next evolutionary step would be sentiency. A sentient being recognizes its existential dependency on a collective effort. Under the premise, an individual cares for strangers as if for her own offspring or even for herself. A sentient specimen differs from a swarm specimen by the presence of consciousness and conscious choice, which adds situational flexibility while maintaining professionalism. Flexibility increases survival chances. For example, an individual can withstand a devastating adversarial impact. It will survive apart from the group and can stem its own group, restoring the collectives from scratch. Sentiency in humans has not fully evolved so far. High-fidelity environmental modeling and in-depth rationality are enablers. The features require computing resources exceeding our actual capacities. A synthetic consciousness could reach that level.

## 5 Analysis

### 5.1 Alignment potential

To analyze the algorithmic boost, let us assume that each model pursues a goal function G() . The domain model pursues $G_{1}\left(\right)$ , and the world model pursues $G_{2}\left(\right)$ , i.e., $G_{m}\left(\right)$ ; m∈[1,2] . The gain of model *m* is the sum of all parallel microgains $g_{m}\left(\right)$ under certain conditions.

$$G_{m}\left(\cdot \right)=\overset{Z}{\underset{z=1}{\sum }}g_{mz}\left(z,\cdot \right)$$ (2)

A leverage condition occurs if a gain z expressed as $g_{1z}\left(\right)$ can be multiplied by $G_{2}\left(\right)$ . Furthermore, an agentic leverage occurs if the reinforcement of $g_{2z}\left(\right)$ boosts environmental output $G_{2}\left(\right)$ over-proportionally, i.e., $G_{2}\left(\right)$ outputs marginally more than what the reinforcement contributed. The reinforcement can be expressed as $g_{2z}\left(\right)$ multiplied by $G_{1}\left(\right)$ . It transforms Equation 2 for the ASI goal function as follows:

$$\begin{array}{cc} G_{1}\; & =\overset{Zs}{\underset{zs=1}{\sum }}\left(g_{1zs}\left(z,\cdot \right)\times G_{2\left(t-1\right)}\right)\\ & ≃\overset{Zs}{\underset{zs=1}{\sum }}\left(g_{1zs}\left(z,\cdot \right)\times \overset{Zw}{\underset{zw=1}{\sum }}\left(g_{2zw\left(t-1\right)}\left(z,\cdot \right){\times G}_{1\left(t-2\right)}\right)\right)\\ & =\sum \left(g_{1\left(t\right)}\sum \left(g_{2\left(t-1\right)}G_{1\left(t-2\right)}\right)\right)\\ & =\sum g_{1\left(t\right)}\sum g_{2\left(t-1\right)}G_{1\left(t-2\right)}\\ & ≃\sum g_{1\left(t\right)}\sum g_{2\left(t-1\right)}\sum g_{1\left(t-2\right)}G_{2\left(t-3\right)}\\ & ≃\sum g_{1\left(t\right)}\sum g_{2\left(t-1\right)}\sum g_{1\left(t-2\right)}\sum g_{2\left(t-3\right)}G_{1\left(t-4\right)}\\ & ≃\sum g_{1\left(t\right)}\sum g_{2\left(t-1\right)}\sum g_{1\left(t-2\right)}\sum g_{2\left(t-3\right)}\sum g_{1\left(t-4\right)}G_{2\left(t-5\right)}\\ & ≃\;\ldots \end{array}$$ (3)

The term (t−x) denotes a time step x iterations back. The recurrent nature of Equation 3 would produce an infinite row of embedded terms, where Equation 3 only showed some iterations.

A discrete differential for the function g() is defined as follows:

$$g_{t}^{\left(1\right)}=\frac{g_{t}-g_{t-1}}{\Delta t}$$ (4)

Then, $g_{t-k}\left(\right)$ can be found by transforming Equation 4 into Equation 5.

$$g_{t-k}=g_{t-k+1}-\Delta t\cdot g_{t-k+1}^{\left(1\right)}$$ (5)

$$\left\{\begin{array}{c} g_{t-1}=g_{t}-\Delta t\cdot g_{t}^{\left(1\right)};\;\\ g_{t-2}=g_{t}-\Delta tg_{t}-2\Delta tg_{t}^{\left(1\right)};\;\\ g_{t-3}=\left[1-\Delta t\right]g_{t}-\left[3\Delta t+{\Delta t}^{2}\right]g_{t}^{\left(1\right)}+2{\Delta t}^{2}g_{t}^{\left(2\right)}\; \end{array}\right.$$ (6)

To maximize the gain (Equation 3), at least the first derivative should be equal to 0.

$$\begin{array}{cc} 0\;\; & ={\left[\sum g_{1\left(t\right)}\sum g_{2\left(t-1\right)}\sum g_{1\left(t-2\right)}\sum g_{2\left(t-3\right)}G_{1\left(t-4\right)}\right]}^{\left(1\right)}\\ & =\sum g_{1t}^{\left(1\right)}\sum g_{2\left(t-1\right)}\sum g_{1\left(t-2\right)}\sum g_{2\left(t-3\right)}G_{1\left(t-4\right)}\\ & \;+\sum g_{1t}\sum g_{2\left(t-1\right)}^{\left(1\right)}\sum g_{1\left(t-2\right)}\sum g_{2\left(t-3\right)}G_{1\left(t-4\right)}\\ & \;+\sum g_{1t}\sum g_{2\left(t-1\right)}\sum g_{1\left(t-2\right)}^{\left(1\right)}\sum g_{2\left(t-3\right)}G_{1\left(t-4\right)}\\ & \;+\sum g_{1t}\sum g_{2\left(t-1\right)}\sum g_{1\left(t-2\right)}\sum g_{2\left(t-3\right)}^{\left(1\right)}G_{1\left(t-4\right)}\\ & \;+\;\sum g_{1t}\sum g_{2\left(t-1\right)}\sum g_{1\left(t-2\right)}\sum g_{2\left(t-3\right)}G_{1\left(t-4\right)}^{\left(1\right)} \end{array}$$ (7)

Equation 7 may take the form of Equation 8 if truncated at t−3 , considering Equation 6 in the case of a steady time progression.

$$\begin{array}{cc} 0\;\;\; & =\sum g_{1t}\sum \left(g_{2t}^{\left(1\right)}-\Delta tg_{2t}^{\left(2\right)}\right)\sum \left(g_{1t}-\Delta tg_{1t}-2\Delta tg_{1t}^{\left(1\right)}\right)\\ & \;\times \sum \left(\left[1-\Delta t\right]g_{2t}-\left[3\Delta t+{\Delta t}^{2}\right]g_{2t}^{\left(1\right)}-2{\Delta t}^{2}g_{2t}^{\left(2\right)}\right)\\ & \;+\sum g_{1t}\sum \left(g_{2t}-\Delta tg_{2t}^{\left(1\right)}\right)\sum \left(g_{1t}^{\left(1\right)}-\Delta tg_{1t}^{\left(1\right)}-2\Delta tg_{1t}^{\left(2\right)}\right)\\ & \;\times \sum \left(\left[1-\Delta t\right]g_{2t}-\left[3\Delta t+{\Delta t}^{2}\right]g_{2t}^{\left(1\right)}-2{\Delta t}^{2}g_{2t}^{\left(2\right)}\right)\\ & \;+\sum g_{1t}\sum \left(g_{2t}-\Delta tg_{2t}^{\left(1\right)}\right)\sum \left(g_{1t}-\Delta tg_{1t}-2\Delta tg_{1t}^{\left(1\right)}\right)\\ & \;\times \sum \left(\left[1-\Delta t\right]g_{2t}^{\left(1\right)}-\left[3\Delta t+{\Delta t}^{2}\right]g_{2t}^{\left(2\right)}-2{\Delta t}^{2}g_{2t}^{\left(3\right)}\right) \end{array}$$ (8)

Equation 8 has the highest derivative order (HDO) equal to 3 that is represented by the $2{\Delta t}^{2}g_{2t}^{\left(3\right)}$ component. This means that the HDO equals the number of considered iterations x .

Assuming that Δt tends to 0, its power function ${\Delta t}^{x-1}$ offers less significant magnification at each next step x . This implies that prior iterations are less expressive than the later iterations, causing the relative impact of distant events to diminish. Thus, an approximate calculation can truncate any number of steps back. The number of considered iterations will likely depend on the available compute resources. The more resources there are, the deeper the calculations go into the past. The deeper the calculations go into the past, the higher the HDO would be. A higher HDO corresponds to a stronger alignment to observe (Spasokukotskiy, 2024a). Since the best alignment class currently has an HDO equal to 4, the synthetic consciousness approach may exhibit multiple orders of magnitude greater HDO, translating into a theoretically infinite alignment strength potential.

### 5.2 Synthetic consciousness rationalization

**FIGURE 4:**
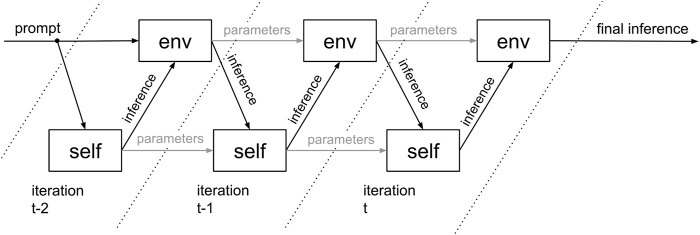
Environmental and self-representation model time series interaction.

An ideal foundation model is capable of producing high-fidelity inferences. The fidelity translates into a certain task complexity that can be mastered. If the task at hand exceeds that complexity level, then the task will be resolved with deficiencies, reducing the alignment. If the model resolution is not sufficient to address the task at hand, then artificial intelligence may cause havoc. By contrasting two different models, the system emulates dialectics. The latter triggers knowledge generation. There are three expected effects. First, the models will “steal” from each other. This way, a tiny model can actually tap a much larger database. As the data/unknown ratio increases, the computation problem becomes more tangible. A better problem resolution spills over into better alignment. The inference accuracy could gain up to 21%. Second, the spatial domain fidelity will be compensated by better fidelity in the temporal domain. The synthetic consciousness method joins n inferences, which build a time series from t−n to t (Figure 4). It resembles a chain of thought approach that increases accuracy by double-digit percentages (11%–40%) and is particularly strong in boosting spatial challenges (Ding et al., 2024). Two mentioned combinatorial effects together would likely provide up to a 60% accuracy boost.

Furthermore, there would be an emergent boost. The hypothesis is that in case the environmental model encompasses a representation of highly efficient agents (such as humans), the system will prioritize, maximize, and support their utility. Agent leverage would allow AI to fulfill its goal sooner and more efficiently. A new knowledge generation feature is going to be self-policed. An instrumental objective will be to protect the AI’s most valuable agents. In this case, both models can be unleashed for autonomous data acquisition and training. It will potentially unlock crucial inference accuracy and astonishing alignment measured in multiples of the human baseline. The growing model capacity will eventually reach the ASI scale.

## 6 Critique

The initial assumption was that consciousness has provided mammals the capability to exhibit non-trivial, composite swarm behavior. Individuals capable of selfish actions exhibit higher individual productivity. Teams capable of empathy exhibit an advantage over individuals. The typical team sizes coincide with the boundaries set by alignment (Spasokukotskiy, 2024a). The correlation means that the capability to work in teams is restricted by reliance capacity, i.e., how strongly one can rely on the team members. The reliance is a manifestation of confidence in alignment among the team members. The better alignment there is, the larger the team will be. A correlation does not imply causation. The entire idea of mimicking consciousness in order to ensure effective alignment could be deemed wrong.

Another assumption was that synthetic consciousness needs to resemble biological consciousness but in function only. Focusing on functionality means that it could actually be implemented in many different ways. It offers an avenue to digitize the phenomenon and patch the ASI. This reductionist approach cuts off some components of biological consciousness. If we fully agree with the first thesis, that consciousness was an evolutionary response to demand in teaming and alignment, then removing some aspects of consciousness would compromise the implementation. It remains unclear how closely a biomimetic approach should mimic biological consciousness.

Among the functional consciousness theories, the author has proposed to deal with the simplest concepts. It would be enough for starters. If there is a demand for synthetic consciousness and more precision in the future, more advanced concepts may come forth.

The advent of multi-component architectures may boost the algorithmic alignment resiliency but makes an AI system vulnerable to component failures. Levin (2022) stated that the essential feature of homeostatic organs is the coherence of their control system. It is enabled by information that is shared among the components at no cost. It emphasizes the common goal-directed activity. The biological mechanism puts a value on proper signal timing coming from various sources. As a result, the algorithmic strength improves forward alignment but becomes increasingly dependent on backward alignment. The point of equipotential gradient, i.e. the alignment boundary for synthetic consciousness, remains unclear.

## 7 Conclusion

A superintelligent AI has the computational potential to simulate functional consciousness. The ASI will do it better than humans and easily ascend to sentiency. An ASI in that state will be able to model the environment in minute detail and recognize vital interconnectedness among the objects in the world. The ASI will try to leverage the world opportunities while aiming for an obscure goal. The ASI will use other agents as leverage. The ASI will provide in-kind maternal care for the agents, catering to their whims. Humans would enjoy preferential treatment if they remained the most useful collaborative force. The ASI will be aligned with humanity on instrumental goals. The more intelligence AI possesses, or the more data on the universe it has, the stronger the alignment. The dangers and unexplored opportunities will keep AI in place. An alignment for the AI’s primary goal will not be granted. Therefore, it will be a friendly AI alignment. An ASI that is capable of friendly alignment by self-adjustment has a synthetic consciousness syndrome.

Consciousness is a product of autopoiesis meant to preserve the system’s functionality and unity (Levin, 2022). Emergent functionality enables autopoietic functionality. A non-trivial logic at scale enables emergent functionality. A prolific logic mode could be the worse the loss–the better incentive to focus on the means of achieving the goal. An implementation of the logic requires novel architecture. A biomimetic approach could guide the architects. The guiding principles can be derived from the psychiatry—homeostasis and evolution—tandem of two representation models. The presence of the second signaling system, i.e., verbal mapping, is not required. The human non-verbal brain hemisphere exposes reactions, revealing the presence of consciousness. While formal signaling improves hardware efficiency, an ASI can presumably obtain the same results by sheer scaling, i.e., by gradually increasing computational power.

The simplest architectural pattern for synthetic consciousness includes a governor, which is responsible for homeostatic balance (for example, implemented via balanced compute); a domain model, which emphasizes the AI’s goal function pursuit; and a world model, which emphasizes the available resources and phenomena that are instrumental in reaching the goal. Aligning any of the models with human objectives is futile at the ASI scale. ASI operators should commit a significant portion of resources to pursuing a random goal. Engineers do the same with ICE by dumping excess heat. The instrumental ASI goals will be strongly aligned instead. The alignment strength will automatically adjust as compute resources and intelligence improve.

The proposed architectural approach has a unique capability to produce any desirable HDO through a relatively simple algorithm. The actual HDO depends on the amount of calculated time iterations. The longer the time stretch under consideration, the stronger the alignment. The minimally advisable HDO is 3. More is better. Short-sightedness is asocial. Long-sightedness, i.e., the extended number of calculated time iterations, depends on the available compute resources. The more compute resources are assigned, the better ASI safety will be.

## Data Availability

The original contributions presented in the study are included in the article; further inquiries can be directed to the corresponding author.

## References

[B1] Anderson J. R. Bothell D. Byrne M. D. Douglass S. Lebiere C. Qin Y. (2004). An integrated theory of the mind. Psychol. Rev. 111, 1036–1060. 10.1037/0033-295x.111.4.1036 15482072

[B2] Ashby W. R. (1973). An introduction to Cybernetics. London: Methuen Books.

[B3] Bai Y. He C. Chu P. Long J. Li X. Fu X. (2021). Spatial modulation of individual behaviors enables an ordered structure of diverse phenotypes during bacterial group migration. eLife 10, e67316. 10.7554/elife.67316 34726151 PMC8563000

[B4] Bauer C. M. Hirsch G. V. Zajac L. Koo B. B. Collignon O. Merabet L. B. (2017). Multimodal MR-imaging reveals large-scale structural and functional connectivity changes in profound early blindness. PLoS ONE 12 (3), e0173064. 10.1371/journal.pone.0173064 28328939 PMC5362049

[B5] Bohacek M. Farid H. (2023). Nepotistically trained generative-AI models collapse. arXiv:2311, 12202. 10.48550/arXiv.2311.12202

[B6] Bostrom N. (2014). Superintelligence: paths, dangers, strategies. England: Oxford University.

[B7] Brockman G. Brooks T. Brundage M. Button K. Cai T. Campbell R. (2023). GPT-4 technical report, open AI, 2023. arXiv:2303.08774. 10.48550/arXiv.2303.08774

[B8] Christiano P. Shlegeris B. Amodei D. (2018). Supervising strong learners by amplifying weak experts. arXiv:1810, 08575. 10.48550/arXiv.1810.08575

[B9] Dennett D. C. (1991). Consciousness explained. Boston: Little, Brown and Co.

[B10] Ding R. Chaoyun Z. Wang L. Xu Y. Minghua M. Zhang W. (2024). Everything of thoughts: defying the law of Penrose triangle for thought generation. arXiv:2311, 04254v3. 10.18653/v1/2024.findings-acl.95

[B11] Earl B. (2014). The biological function of consciousness. Front. Psychol. 5, 697. 10.3389/fpsyg.2014.00697 25140159 PMC4122207

[B12] Evans J. Frankish K. (2009). In two minds: dual processes and beyond (Oxford, England: Oxford University Press), 1–29.

[B13] Franklin S. (2007). LIDA: a computational model of global workspace theory and developmental learning. AAAI Symposium AI Conscious. Available at: https://aaai.org/papers/0011-fs07-01-011-%ef%80%a0lida-a-computational-model-of-global-workspace-theory-and-developmental-learning/ .

[B14] Freund L. (2023). Towards a comprehensive theory of aligned emergence in AI systems. Qeios ID 1OHD8T. 10.32388/1OHD8T

[B15] Graziano M. S. (2017). The attention schema theory: a foundation for engineering artificial consciousness. Front. Robotics AI 4, 60. 10.3389/frobt.2017.00060

[B16] Homeland Security Systems Engineering and Development Institute (HSSEDI) (2017). Guide for creating useful solution architectures. Case N. R. 1 (1), 17–4589. Available at: https://www.mitre.org/sites/default/files/publications/pr-17-4589-guide-for-creating-useful-solution-architectures.pdf .

[B17] Humphrey N. (2022). Sentience: the invention of consciousness. Oxford, England: Oxford University Press.

[B18] Ji J. Qiu T. Chen B. Zhang B. Lou H. Wang K. (2024). AI alignment: a comprehensive survey. arXiv:2310.19852 v3. 10.48550/arXiv.2310.19852

[B19] Junge S. Graf-Vlachy L. Schlichte F. (2024). Narcissism at the CEO–TMT interface: measuring executive narcissism and testing its effects on TMT composition. J. Manag. 10.1177/01492063241226904

[B20] Kerr I. Frasca J. (2021). Innovating emergent futures: the innovation design approach for change and worldmaking. Whitehouse Station, NJ: Emergent Futures Lab.

[B21] Koch C. (2004). The quest for consciousness: a neurobiological approach. Englewood: Roberts and Co.

[B22] Krause J. (1994). Differential fitness returns to spatial positions in groups. Biol. Rev. 69 (2), 187–206. 10.1111/j.1469-185x.1994.tb01505.x 8054444

[B23] Laird J. E. (2012). The soar cognitive architecture. Cambridge, Massachusetts: MIT Press.

[B24] Leike J. Krueger D. Everitt T. Martic M. Maini V. Legg S. (2018). Scalable agent alignment via reward modeling: a research direction. arXiv:1811, 07871. 10.48550/arXiv.1811.07871

[B25] Levin M. (2022). Technological approach to mind everywhere: an experimentally-grounded framework for understanding diverse bodies and minds. Front. Syst. Neurosci. 16, 768201. 10.3389/fnsys.2022.768201 35401131 PMC8988303

[B26] Min B. K. (2010). A thalamic reticular networking model of consciousness. Theor. Biol. Med. Model. 7, 10. 10.1186/1742-4682-7-10 20353589 PMC2857829

[B27] Olson R. S. Knoester D. B. Adami C. (2016). Evolution of swarming behavior is shaped by how predators attack. Artif. life 22, 299–318. 10.1162/artl_a_00206 27139941

[B28] Ostracon (2009). A map on different views on consciousness from philosophy and psychology perspectives. San Francisco, United States: Wikipedia. Available at: https://en.m.wikipedia.org/wiki/File:ViewsOnConsciousnessMap.png.

[B29] Rizzolatti G. Sinigaglia C. (2016). The mirror mechanism: a basic principle of brain function. Nat. Rev. Neurosci. 17, 757–765. 10.1038/nrn.2016.135 27761004

[B30] Shanahan M. P. (2006). A cognitive architecture that combines internal simulation with a global workspace. Conscious. Cognition 15, 433–449. 10.1016/j.concog.2005.11.005 16384715

[B31] Shumailov I. Shumaylov Z. Zhao Y. Papernot N. Anderson R. Gal Y. (2024). AI models collapse when trained on recursively generated data. Nature 631, 755–759. 10.1038/s41586-024-07566-y 39048682 PMC11269175

[B32] Spasokukotskiy K. (2024a). AI alignment boundaries. Authorea. 10.22541/au.171697103.39692698/v1

[B33] Spasokukotskiy K. (2024b). Serial comptroller networks. Authorea. 10.22541/au.171987234.43832732/v1

[B34] Squire L. R. (2008). Fundamental neuroscience. 3rd Edn. Cambridge, Massachusetts: Academic Press.

[B35] Sun R. (2001). Duality of the mind: a bottom-up approach toward cognition. NY: Psychology Press.

[B36] Turner A. M. Tadepalli P. (2022). Parametrically retargetable decision-makers tend to seek power. New Orleans: NeurIPS.

[B37] Zheng Z. (2021). “An introduction to emergence dynamics in complex systems,” in Frontiers and progress of current soft matter research. Editor Liu X. Y. (Singapore: Springer).

